# Influence of breakfast and meal frequency in calcium intake among pregnant adolescents

**DOI:** 10.1111/mcn.13034

**Published:** 2020-06-08

**Authors:** Maira Pinho‐Pompeu, Daiane Sofia Morais Paulino, Fernanda Garanhani Surita

**Affiliations:** ^1^ Department of Obstetrics and Gynecology University of Campinas Campinas Brazil

**Keywords:** breakfast frequency, calcium intake, dairy products, diet records, meal frequency, pregnancy in adolescence

## Abstract

Calcium is an essential micronutrient that plays a role in growing and pregnancy, and its necessity is increased during pregnancy in adolescence. Thus, the aim of the study is to describe the daily calcium intake and its associations with dietetic habits, sociodemographic data and perinatal outcomes among pregnant adolescents. A prospective cohort study was conducted among primiparous adolescents who started prenatal care before 20 weeks of gestation. Sociodemographic data, weight and height, 24‐h dietary recall (24hRec) and perinatal outcomes were collected over four meetings (three during pregnancy and one in puerperium). All 24hRecs were analysed by the Nutrition Data System for Research (NDSR)® programme, and descriptive analysis and univariate and multivariate logistic regression were done. A total of 150 pregnant adolescents were included, with a mean of daily calcium intake of 659.9 mg (50% of recommended intake). Adolescents who ate more than three meals per day (89.3%), and ate breakfast every day (69.3%), were shown to have higher daily calcium intake, odds ratio (OR CI 95%) of 3.4 (1.0, 11.0) and 16.8 (1.0, 302.1), respectively. No correlation was observed between calcium daily intake and sociodemographic data or perinatal outcomes. Dairy products were the foods that mostly contributed to achieving recommended daily calcium intake. In our cohort, pregnant adolescents had a low daily calcium intake. They should be advised to eat more than three meals per day, eat breakfast in particular, increase the consumption of calcium rich‐foods, such as dairy products and green leafy vegetables, and consider calcium supplementation.

**Key messages**

- Pregnant adolescents have a daily intake of calcium lower than the recommendation of 1,300 mg/day, which can increase the risk of adverse perinatal outcomes.
- Eating more than three meals/day and having the habit of eating breakfast increases the chances of pregnant adolescents achieving calcium recommendation.
- Milk and dairy products are the main calcium dietary sources among pregnant adolescents.
- All pregnant adolescents should be advised to increase their daily calcium intake by ingesting calcium rich‐foods such as milk, dairy products and green leafy vegetables.
- Health professionals should be able to identify pregnant women with inadequate calcium intake, and calcium supplementation should be considered to pregnant adolescents.

## 1 INTRODUCTION

Calcium is an essential nutrient related to bone mineralisation and the maintenance of cell membranes. The best sources of calcium are dairy products, mainly milk. Calcium can also be derived from leafy green vegetables, fish, nuts and fortified foods (de Assumpção, Dias, de Azevedo Barros, Fisberg, & de Azevedo Barros Filho, 2016; Mousa et al., 2019).

During pregnancy, for adequate foetal development and good perinatal outcomes, calcium demand is increased (Mousa et al., 2019). It is advised that pregnant women have an intake of 1,200 mg/day of calcium; however, for women with low daily calcium intake (<1 g/day), supplementation of 0.3–2.0 g/day is recommended (Institute of Medicine & Food and Nutrition Board, 2011; Mousa et al., 2019). The average calcium requirement of the foetus is 60 mg/day by week 24 of the pregnancy and increases with foetal growth, achieving a calcium transfer rate of 300–350 mg/day in the third trimester of pregnancy.

When calcium availability is insufficient, maternal skeleton mineral metabolism mobilises calcium from the mother's skeleton, creating maternal‐foetal competition (Kovacs, 2016). Calcium deficiency during pregnancy can increase the risk of pre‐eclampsia, preterm birth and haemolysis, elevated liver enzymes, and low platelet count (HELLP) syndrome (Hofmeyr, Lawrie, Atallah, & Torloni, 2018; Mousa et al., 2019). Moreover, when calcium is deficient, women are more likely to have osteoporosis and hip fractures in the future (Kovacs, 2016; Mousa et al., 2019).

Peak growth velocity occurs through adolescence, especially bone mass, which increases 40‐fold from birth to the second decade of life (de Assumpção et al., 2016; Wallace, 2019). When pregnancy occurs during adolescence, a maternal‐foetal competition for nutrients is amplified with the purpose of guaranteeing the adequate development of both, the adolescent and foetus (Wallace, 2019). Consequently, the daily calcium intake necessity in a pregnant adolescent (1,300 mg/day) is higher than that of pregnant adult women (1,000 mg/day) (Institute of Medicine & Food and Nutrition Board, 2011).

Among Brazilians female adolescents, the average daily calcium intake is 540.7 mg, which is lower than the average found in Brazilians male adolescents (692.3 mg; de Assumpção et al., 2016) but higher than Brazilian adult population (505 mg; Balk et al., 2017). Pregnant women of low‐ and middle‐income countries have lower average calcium intake (647.6 mg/day) compared to pregnant women of high‐income countries (948.3 mg/day) (Cormick et al., 2019).

Worldwide, 17 million girls under the age of 19 give birth annually. As a result, pregnancy in adolescence is considered a health problem, especially in developing countries (United Nations Population Fund, 2013). Brazil has a pregnancy rate of 68.4 per 1,000 adolescents, and 434 thousand babies are born to adolescents aged 15 to 19 and 29 thousand to adolescents aged 10 to 14 years. Unplanned pregnancies represent 66% of all pregnancies among Brazilian adolescents (The Lancet, 2020).

Maternal nutritional status and dietary intake can reduce the risk of complications (Wallace, 2019). Thus, this study aims to describe the daily calcium intake and its associations with dietetic habits, sociodemographic data and perinatal outcomes among pregnant adolescents.

## 2 METHODS

### 2.1 Setting

This study is part of a larger prospective cohort study conducted at the Women's Hospital Prof. Dr. Jose Aristodemo Pinotti (CAISM), University of Campinas, Brazil, between August 2014 and February 2017, from a convenience sample taken from an outpatient clinic. CAISM is located in Campinas, State of São Paulo, and is one of the largest metropolitan regions of Brazil. The hospital is a referral centre in high‐risk obstetrics, with over 30 years of experience, and 3,000 deliveries per year. CAISM also has an interdisciplinary and multiprofessional team and a specialised outpatient clinic for adolescents, with an average of 150 prenatal care for pregnant adolescents per year.

### 2.2 Sample size

This is a secondary analysis of a study that aimed to evaluate the anthropometric and dietetic profile of pregnant adolescents from the first trimester of pregnancy to the puerperium. Thus, the sample size was calculated according to the prevalence of adequate versus overweight and obese body mass index (BMI) and the prevalence of inadequate plus excessive weight gain in pregnant adolescents (Harper, Chang, & MacOnes, 2011). Considering a 5% significance level, and the difference between the prevalence and 20% loss to follow‐up, the sample size was calculated on a minimum of 138 subjects. Therefore, the sample size was not calculated for dietary analyses.

### 2.3 Participants

Primiparas women under 19 years of age were invited to participate in the study. Adolescents who started prenatal care before 20 weeks of gestational age were excluded to analyse anthropometrics changes that have occurred since the beginning of their pregnancies. All adolescents and their legal guardians signed an informed assent and consent form before their admission to the study.

As bioelectrical impedance analysis (BIA) was applied in the larger study, adolescents with contraindications for BIA were excluded (heart diseases, implanted cardiac defibrillators, pacemakers, prostheses or metal implants, amputations or use of corticosteroids).

### 2.4 Data collection and follow‐up

Data collection occurred at four points, one point in each trimester of pregnancy and one point at puerperium.

At the first point, sociodemographic data (age, ethnicity, have or not have a partner, and currently in school), height, self‐reported pre‐pregnancy weight, age at menarche and gestational age at the first prenatal care visit were collected using a standardised and pre‐tested questionnaire. Age at menarche was split (prior to 12 years and at 12 years or above); early menarche is associated with early sexual initiation, early pregnancy and with adverse pregnancy outcomes (Ibitoye, Choi, Tai, Lee, & Sommer, 2017; Li et al., 2017).

At the last follow‐up point, gestational weight gain (GWG) and obstetric history (anaemia, diabetes, hypertensive disorders, delivery mode, gestational age at delivery, newborn weight and Apgar score) were collected from the medical records.

During all data collections, 24‐h dietary recall (24hRec) and anthropometric assessment by weight were taken.

### 2.5 Dietetic assessment

To calculate the dietary micronutrient intake, a 24hRec was applied in three points of pregnancy and postpartum, totalling four 24hRecs per patient. To fill in the 24hRecs, pregnant adolescents were asked to recall the foods consumed over the previous day and their quantity in household measures. Vitamin and mineral supplements were also included.

All 24hRecs were quantified to convert the amounts of foods mentioned into household measures of grams or millilitres (Fisberg & Slater Villar, 2002; Pinheiro, Lacerda, Benzecry, Gomes, & Costa, 2001). 24hRecs quantities were then transferred to the Nutrition Data System for Research (NDSR®) programme, a dietary analysis programme that provides the quantity of macro‐ and micronutrient intake, the number of meals per day and the name of each meal.

From the number of meals per day, eating frequency was categorised into two groups; adolescents who ate three or more meals per day in all 24hRecs, and adolescents who ate less than three meals per day in all 24hRecs.

To calculate the relationship between breakfast and daily calcium intake, breakfast was considered as all first meals of the day composed of dairy products, bread or cereals and/or fruit. Moreover, breakfast was classified into three groups: adolescents who ate breakfast in all 24hRecs (every day), adolescents who ate breakfast at least in one 24hRecs but not in all (sometimes) and adolescents who did not eat breakfast in any 24hRecs (never).

To calculate foods that contributed the most to adolescents achieving the recommended daily calcium intake, all foods mentioned in the 24hRecs were first categorised according to the type of food (milk, rice, bread or fruit) and then classified according to processing of the food into four groups: unprocessed or minimally processed food, culinary ingredient, processed food and ultra‐processed food (Monteiro, Levy, Claro, de Castro, & Cannon, 2010).

### 2.6 Anthropometric assessment

According to the Institute of Medicine (2009), BMI is calculated as the weight in kilogrammes divided by the height in metres squared. An earlier analyse carried out in our cohort found no statistical differences between the use of self‐reported pre‐pregnancy weight or at the first prenatal care visit to classify BMI and GWG among pregnant adolescents (Pinho‐Pompeu et al., 2019). Based on this, self‐reported pre‐pregnancy weight was assumed for analysis. Pre‐pregnancy BMI was measured by self‐reported pre‐pregnancy weight and height, routinely measured at the first prenatal care visit by locally trained professional nursing staff. To obtain the GWG, the self‐reported pre‐pregnancy weight was subtracted from the weight at delivery, and when this information was missing, the weight at the last prenatal care visit was used. Pre‐pregnancy BMI was classified according to the World Health Organization (WHO) recommendation for adult pregnant women, which was considered a better method of assessing the nutritional status of pregnant adolescents (Pinho‐Pompeu et al., 2019). The BMI classification according to the WHO is as follows: underweight (BMI < 18.5 kg/m^2^), healthy weight (BMI = 18.5–24.9 kg/m^2^), overweight (BMI = 25.0–29.9 kg/m^2^) and obese (BMI ≥ 30.0 kg/m^2^). The WHO's BMI classification is also used by the Institute of Medicine to recommend and classify GWG into “inadequate,” “adequate” and “excessive” (Institute of Medicine, 2009).

### 2.7 Statistical analysis

Frequency analysis of the categorical variables and descriptive analysis of the numerical variables were undertaken to describe the sample profile. Univariate and multivariate logistic regressions (with stepwise criteria of variable selection) were used to correlate categorical variables and low daily calcium intake. For logistical regression, the daily calcium intake was categorised according to the group median (600 mg/day), because there is no minimum recommendation of daily calcium intake for pregnant adolescents in the literature and only 4% of adolescents had a calcium intake above the recommendation of 1,300 mg/day (Institute of Medicine & Food and Nutrition Board, 2011).

The significance level for the statistical tests was 5%, and the statistical software used for the analysis was Stata version 14.0 for Windows.

All items of the Strengthening the Reporting of Observational Studies in Epidemiology consensus were followed (von Elm et al., 2014).

### 2.8 Ethical considerations

This study was approved by the Institutional Review Board of the University of Campinas, Brazil (CAAE report: 35521414.2.0000.5404).

## 3 RESULTS

A total of 150 pregnant adolescents with a mean age of 15.5 (standard deviation; SD 1.3) years and mean age at menarche of 11.6 (SD 1.3; Table 1), were included on study. The mean gestational age at the first prenatal care visit was 14.9 (SD 3.6) weeks.

**TABLE 1:** Sociodemographic, anthropometric and dietetic characteristics of pregnant Brazilian adolescents (*n* = 150)

	*N*	%
Age group		
<15 years	42	28.0
≥15 years	108	72.0
Skin colour		
White	81	55.9
Non‐White	64	44.1
Missing	5	3.3
Partner		
With	124	83.8
Without	24	16.2
Missing	2	1.3
Student		
Yes	96	64
No	54	36
Menarche (years)		
≥12	79	52.7
<12	71	47.3
Pre‐pregnancy BMI^a^		
Low weight	24	16.0
Normal weight	99	66.0
Overweight	18	12.0
Obese	9	6.0
Gestational weight gain^a^		
Inadequate	37	32.2
Adequate	40	34.8
Excessive	38	33.0
Eating frequency		
≤3 meals per day	16	10.7
>3 meals per day	134	89.3
Habit of eating breakfast		
Every day	104	69.3
Sometimes	39	26.0
Never	7	4.7

Abbreviation: BMI, body mass index.

^a^ Classified according to the Institute of Medicine recommendation, according to the pre‐pregnancy BMI (Institute of Medicine, 2009).

The average pre‐pregnancy weight was 55.9 (SD 11) kg with a pre‐pregnancy BMI mean of 22.4 (SD 4.6) kg/m^2^ and GWG mean of 13.2 (SD 5.6) kg. More anthropometric data are presented in Table 1.

Among the perinatal outcomes, 14.7% (*n* = 16) of adolescents had gestational diabetes, 47.7% (*n* = 53) had anaemia and 10.9% (*n* = 13) had some type of hypertensive disorder during pregnancy. Vaginal birth was observed in 60.8% (*n* = 73) of deliveries, 87.4% (*n* = 104) of newborns were born at ≥37 weeks of gestational age, 9.6% (*n* = 11) of newborns were born weighing <2,500 g and all newborns had Apgar scores greater than 7 at the fifth minute.

A daily calcium intake above the recommendation (1,300 mg/day) was observed in 4% (*n* = 6) of adolescents. The average daily calcium intake was 659.9 (SD 335.0) mg, with a minimum of 179.9 mg, a median of 604.6 mg and maximum of 2,017.4 mg. Isolated calcium supplementation was not observed, but 6% (*n* = 5) of adolescents were taking multiple vitamin and mineral supplements for adult pregnant women.

The habit of having breakfast every day was observed in 69.3% (*n* = 104) of the adolescents, 4.7% (*n* = 7) were not in the habit of having breakfast, 89.3% (*n* = 134) ate more than three meals a day (Table 1). A correlation between daily calcium intake above 600 mg/day, eating frequency (*p* = 0.04), and the habit of eating breakfast (*p* < 0.05) was found by the logistic regression (Table 2). No correlation was observed between calcium daily intake and the other variables.

**TABLE 2:** Logistic regression results for daily calcium intake below 600 mg/day among pregnant Brazilian adolescents (*n* = 150)

	*P* value	Odds ratio	CI 95% OR
Age (years)	0.76	0.96	0.76, 1.23
Skin colour			
White	Reference		
Non‐white	0.47	0.77	0.38, 1.57
Partner			
With	Reference		
Without	0.66	1.22	0.51, 2.93
Student			
Yes	Reference		
No	0.73	0.89	0.46, 1.74
Menarche (years)			
≥12	Reference		
<12	0.62	0.85	0.45, 1.62
Gestational age at 1^th^ prenatal care visit	0.19	1.06	0.91, 1.16
Body mass index			
Low weight	0.82	1.11	0.45, 2.70
Normal weight	Reference		
Overweight	0.14	2.21	0.77, 6.36
Obese	0.87	0.89	0.22, 3.49
Gestational weight gain			
Inadequate	0.54	0.75	0.31, 1.86
Adequate	Reference		
Excessive	0.36	1.52	0.62, 1.02
Eating frequency			
≤3 meals per day	0.04	3.38	1.04, 11.02
>3 meals per day	Reference		
Habit of eating breakfast			
Every day	Reference		
Sometimes	0.86	1.07	0.51, 2.23
Never	<0.05	16.82	1.01, 302.08
Fasting blood glucose (mg/dL)			
<92	Reference		
≥92	0.38	1.63	0.55, 4.86
Haemoglobin (mg/dL)			
≥11	Reference		
10.0–10.9	0.10	0.40	0.14, 1.19
<9.9	0.17	1.87	0.77, 4.54
Hypertension disorders			
No	Reference		
Yes	0.75	0.83	0.26, 2.62
Birth mode			
Vaginal	Reference		
Caesarean	0.18	1.68	0.79, 3.60
Gestational age at birth (weeks)			
≥37	Reference		
<37	0.76	0.84	0.29, 2.49
Newborn weigh (g)			
≥2,500	Reference		
<2,500	0.33	0.53	0.15, 1.92

*Note.* Number of observations used = 150; *n* = 72 with daily calcium intake ≥ 600 mg and *n* = 70 with daily calcium intake <600 mg.

Abbreviations: CI, confidence interval; OR, odds ratio.

Into ultra‐processed foods, yoghurt was a major contributor to dietary calcium intake among pregnant adolescents, contributing to 39.7% of the total calcium intake; milk was also a major contributor with 31.7% of total calcium intake (Table 3).

**TABLE 3:** Contribution of each food processing group to total calcium intake of pregnant Brazilian adolescents (*n* = 150)

	Absolute	Calcium	Carbohydrate	Protein	Fat	Total sugar	Fibre	Sodium
	Kcal/day	% of total intake (mg)	% of total intake (g)	% of total intake (g)	% of total intake (g)	% of total intake (g)	% of total intake (g)	% of total intake (mg)
**1. Unprocessed foods**	**899.4**	**45.2 (300.9)**	**45.6 (300.9)**	**66.3 (52.6)**	**31.2 (25.1)**	**25.1 (11.7)**	**41.3 (5.7)**	**28.9 (992.6)**
*Milk*	94.2	31.4 (209.5)	31.7 (209.5)	7.3 (5.8)	6.9 (5.6)	8.8 (4.1)	0 (0)	2.3 (79.8)
*Plain yoghurt*	0.9 (79.7)	28.1 (187.3)	28.4 (187.3)	6.8 (5.4)	4.1 (3.3)	6.6 (2.6)	0 (0)	2.1 (71.7)
*Green leafy vegetables*	0.2 (4.4)	1.6 (10.9)	1.6 (10.9)	0.4 (0.3)	0 (0)	0.2 (0.1)	2.9 (0.4)	0,2 (5.8)
**2. Processed foods**	**245.8**	**17.8 (118.3)**	**17.9 (118.3)**	**15.9 (12.7)**	**4.2 (3.4)**	**1.7 (0.8)**	**36.9 (5.1)**	**13.6 (466.9)**
*Canned food*	50.4	8.6 (57.0)	8.6 (57.0)	7.7 (6.1)	0.4 (0.3)	0.6 (0.3)	0.6 (4.1)	5.9 (201.4)
*Cheese*	66.7	6.6 (44.1)	6.7 (44.1)	2.0 (1.6)	2.2 (1.8)	0.06 (0.03)	0 (0)	1.5 (53.2)
**3. Ultra‐processed foods**	**711.2**	**36.6 (243.8)**	**36.9 (243.8)**	**17.7 (14.0)**	**37.3 (30.1)**	**57.1 (26.6)**	**21.8 (3.0)**	**30.1 (1,030.8)**
*Yoghurt*	6.9 (144.9)	39.4 (262.2)	39.7 (262.2)	8.4 (6.7)	5.8 (4.7)	18.2 (8.5)	7.9 (1.1)	2.6 (88.8)
*Chocolate powder*	88.9	9.4 (62.7)	9.5 (62.7)	0.6 (0.5)	0.2 (0.2)	7.5 (3.5)	2.9 (0.4)	0.6 (20.2)
*Sweets*	171.4	8.0 (53.5)	8.1 (53.5)	2.3 (1.8)	5.3 (4.3)	14.6 (6.8)	2.9 (0.4)	1.2 (39.9)
*Soft drinks*	82.7	6.1 (40.8)	6.2 (40.8)	1.1 (0.9)	0.6 (0.5)	21.2 (9.9)	1.4 (0.2)	0.7 (22.8)
**4. Processed culinary ingredients** ^a^	**255.1**	**0.4 (2.6)**	**0.4 (2.6)**	**0.1 (0.06)**	**27.3 (22.0)**	**16.1 (7.5)**	**0 (0)**	**27.4 (938.7)**
**Total**	2111.5	100 (666.1)	100 (659.9)	100 (79.4)	100 (80.6)	100 (46.6)	100 (13.8)	100 (3,428.9)

^a^ Salt, sugar and oil add to cook.

## 4 DISCUSSION

A better daily calcium intake among adolescents who had the habit of eating breakfast, and among adolescents who had the habit of eating more meals per day was found in our cohort. However, these habits were not sufficient for adolescents to achieve the daily calcium intake recommendation, which was observed in only 4% of pregnant adolescents.

In our cohort, only one‐third of pregnant adolescents had the habit of eating breakfast. These data ware in agreement with the dietary behaviours of most adolescent populations worldwide (Hassan, Cunha, da Veiga, Pereira, & Sichieri, 2018; Inchley et al., 2016). In most cultures, breakfast is composed of bread or cereals, dairy food products (mainly milk) and fruits and contributes to approximately 5% of total calcium intake (Hassan et al., 2018; O'Neil et al., 2014). Moreover, studies have shown that skipping breakfast is not only related to the risk of calcium deficiency, and its negatives consequences for pregnancy, but also is related to metabolic health (Monzani et al., 2019; Pot, 2018). Not eating breakfast frequently can impact on weight status, contributing to overweight, obesity and metabolic syndrome (Monzani et al., 2019). In addition, skipping breakfast can also affect cognitive and academic performance among adolescents (Pot, 2018).

Apart from this, 10% of adolescents of our cohort did not have the habit of consuming more than three meals per day. Others studies have also found a higher prevalence of skipping meals (19.1%) among adolescents (boys and girls) and adults, with breakfast being the most frequently skipped meal (Rodrigues et al., 2017; St‐Onge et al., 2017). In addition, a low‐quality diet with low consumption of fruits and vegetables and a high intake of sodium and calories were also associated with skipping meals (Rodrigues et al., 2017). Low meal frequency has been related to higher weight and higher cardiometabolic risk (St‐Onge et al., 2017).

The results call attention to the low daily calcium intake among pregnant adolescents of our cohort (mean of 659.9, SD 335.0 mg). Similar results have been found all over the world among adolescents (Bromage, Ahmed, & Fawzi, 2016; Lappe et al., 2017). In Brazil, studies have shown a mean calcium intake of 618 mg/day for boys and girls, but mean calcium intake was lower among girls (540.7 mg/day; de Assumpção et al., 2016); and only one‐third of pregnant adolescents reached the calcium recommendation, with no differences between daily calcium intake and nutritional status (Sally et al., 2018). However, low calcium intake is not only an adolescent problem. Calcium deficiency is a global problem, especially in low‐ and middle‐income countries (Cormick et al., 2019). In many countries in Asia, Africa and South America, daily calcium intake is less than 600 mg/day in almost the whole population (Balk et al., 2017). These results show us that low calcium intake is not only a pregnant adolescent problem, and calcium supplementation in pregnant women in low‐ and middle‐income countries should be universal (Cormick et al., 2019).

Another important contributor to the daily calcium intake was the quality of diet. In our cohort, the food that contributed most to improved calcium intake in adolescents was ultra‐processed yoghurt (39.7%); however, the type of yoghurt has added sugar and flavourings, and as with all ultra‐processed food, has high percentages of carbohydrate, fat, sugar and sodium along with low percentages of protein and fibre, and therefore should not be recommended as a way to increase daily calcium intake (Monteiro et al., 2010). Milk was the second highest contributor increasing calcium intake, which represented more than 30% of all calcium intake. Dairy products, such as cheese and plain yoghurt, together represented 35.1% of all calcium intake by adolescents and are also important sources of calcium (Institute of Medicine & Food and Nutrition Board, 2011). Chocolate powder and sweets also contributed to increased daily calcium intake (17.6% together) in our cohort, as these foods are enriched with micronutrients; however, these are not natural calcium‐rich foods and similar to ultra‐processed yoghurt and should not be recommended for increasing daily calcium intake. Moreover, higher consumption of ultra‐processed foods has a positive correlation with GWG and neonatal body fat, increasing the risk of adverse perinatal outcomes (Rohatgi et al., 2017). Green leafy vegetables are considered an important source of calcium (Institute of Medicine & Food and Nutrition Board, 2011); however, these represented only 1.6% of all calcium intake.

In recent times, a reduction in the consumption of dairy products has been observed. It is important to highlight that dairy products; when consumed according to appropriate national guidelines, contribute to daily calcium intake and other essential nutrients, especially during the period of bone mass growth of infancy, childhood and adolescence (Marangoni et al., 2019). These findings raise the possibility that adolescents who eat breakfast, have a good meal frequency and eat more dairy products are more likely to achieve calcium recommendations.

Population studies with Brazilian adolescents have shown that unhealthy food intake is common among this population, which usually choose foods based on appearance and taste, cravings, convenience and cost (Maia et al., 2018; Wise, 2015). The weekly frequency of the consumption of treats and ultra‐processed salted foods in adolescents is 3.76 and 3.26 days/week, respectively (Maia et al., 2018). In comparison, vegetables are consumed 3.43 days/week (Maia et al., 2018), and 51.5% of adolescents have a regular intake (≥5 times/week) of milk, with fewer than 30% of adolescents consuming raw and cooked vegetables (≥5 times/week; Azeredo et al., 2015). In addition, population studies have shown that only 47% to 63.5% of adolescents have the habit of having breakfast more than 5 days a week (Azeredo et al., 2015; Maia et al., 2018; Rodrigues et al., 2017), and the habit of having breakfast was associated with greater consumption of milk and dairy products (Rodrigues et al., 2017).

Female adolescents were more likely to present unhealthy eating patterns (*P* < 0.005; Maia et al., 2018) and do not have the habit of heaving breakfast more than 5 days a week (*P* < 0.001; Azeredo et al., 2015). However, pregnancy can have an influence on a healthy lifestyle and the provision of nutritional education is an opportunity to stimulate healthy eating habits. Pregnancy can influence dietary patterns, which could be explain the lower prevalence of skipping meals found in our study cohort (Wise, 2015).

It is essential to highlight that there is no recommendation for the minimum daily calcium intake among pregnant adolescents, and the intake above the recommendation was very low in our cohort (Institute of Medicine & Food and Nutrition Board, 2011). Therefore, the median was considered for analyses, and the results showed that a daily calcium intake above 600 mg/day was better than below that, while still being insufficient. Moreover, using a 24‐h dietary recall to access daily calcium intake can present some limitations, such as recall bias, the inability of a single day's intake to represent the typical diet, the interview occurring after an untypical diet day and collecting differences between interviewers. To reduce bias, some strategies were applied, such as a multiple‐pass questioning method was used, which included questions about frequently “forgotten foods,” 24‐h dietary recalls were applied more than one time, and in different days of the week, and all interviewers were previously trained. Despite that, to our knowledge, this is the first study to evaluate the influence of breakfast and meal frequency on daily calcium intake among pregnant adolescents.

## 5 CONCLUSION

Pregnant adolescents have low daily calcium intake. However, the habit of eating breakfast and eating at least three meals per day increases calcium intake, but it is not sufficient to achieve the daily calcium recommendation. Accordingly, girls should be advised by health professionals to increase their daily calcium intake by eating breakfast daily, increasing eating frequency and including calcium rich‐foods in their diet, such as dairy products and green leafy vegetables. Even with nutrition counselling, dietary calcium intake among pregnant adolescents should be evaluated, and when necessary, calcium supplementation should be considered to guarantee the daily calcium recommendation.

## CONFLICTS OF INTEREST

The authors declare that they have no conflict of interests.

## CONTRIBUTIONS

All authors contributed to the development of the research project. MPP and DSMP contributed to the investigation, data curation and writing—original draft. FGS also contributed to the review, edition and supervision of the original draft.

## References

[mcn13034-bib-0001] de Assumpção, D. , Dias, M. R. M. G. , de Azevedo Barros, M. B. , Fisberg, R. M. , & de Azevedo Barros Filho, A. (2016). Calcium intake by adolescents: A population‐based health survey. Jornal de Pediatria, 92(3), 251–259. 10.1016/J.JPED.2015.09.004 26738890

[mcn13034-bib-0002] Azeredo, C. M. , De Rezende, L. F. M. , Canella, D. S. , Moreira Claro, R. , De Castro, I. R. R. , Luiz, O. D. C. , & Levy, R. B. (2015). Dietary intake of Brazilian adolescents. Public Health Nutrition, 18, 1215–1224. 10.1017/S1368980014001463 25089589 PMC10271650

[mcn13034-bib-0003] Balk, E. M. , Adam, G. P. , Langberg, V. N. , Earley, A. , Clark, P. , Ebeling, P. R. , … Dawson‐Hughes, B. (2017). Global dietary calcium intake among adults: A systematic review. Osteoporosis International: A Journal Established as Result of Cooperation between the European Foundation for Osteoporosis and the National Osteoporosis Foundation of the USA, 28(12), 3315–3324. 10.1007/s00198-017-4230-x PMC5684325 29026938

[mcn13034-bib-0004] Bromage, S. , Ahmed, T. , & Fawzi, W. W. (2016). Calcium deficiency in Bangladesh: Burden and proposed solutions for the first 1000 days. Food and Nutrition Bulletin, 37(4), 475–493. 10.1177/0379572116652748 27307152 PMC5135641

[mcn13034-bib-0005] Cormick, G. , Betrán, A. P. , Romero, I. B. , Lombardo, C. F. , Gülmezoglu, A. M. , Ciapponi, A. , & Belizán, J. M. (2019). Global inequities in dietary calcium intake during pregnancy: A systematic review and meta‐analysis. BJOG : An International Journal of Obstetrics and Gynaecology, 126(4), 444–456. 10.1111/1471-0528.15512 30347499 PMC6518872

[mcn13034-bib-0006] von Elm, E. , Altman, D. G. , Egger, M. , Pocock, S. J. , Gøtzsche, P. C. , & Vandenbroucke, J. P. (2014). The strengthening the reporting of observational studies in epidemiology (STROBE) statement: Guidelines for reporting observational studies. International Journal of Surgery, 12, 1495–1499. 10.1016/j.ijsu.2014.07.013 25046131

[mcn13034-bib-0007] Fisberg, R. M. , & Slater Villar, B. (2002). Manual de receitas e medidas caseiras para cálculo de inquéritos alimentares: Manual elaborado para auxiliar o processamento de dados de inquéritos alimentares. Signus. Retrieved from https://bdpi.usp.br/item/001557478

[mcn13034-bib-0008] Harper, L. M. , Chang, J. J. , & MacOnes, G. A. (2011). Adolescent pregnancy and gestational weight gain: Do the Institute of Medicine recommendations apply? American Journal of Obstetrics and Gynecology, 205, 140.e1–140.e8. 10.1016/j.ajog.2011.03.053 21620365 PMC3164947

[mcn13034-bib-0009] Hassan, B. K. , Cunha, D. B. , da Veiga, G. V. , Pereira, R. A. , & Sichieri, R. (2018). Changes in breakfast frequency and composition during adolescence: The Adolescent Nutritional Assessment Longitudinal Study, a cohort from Brazil. PLoS ONE, 13(7), e0200587 10.1371/journal.pone.0200587 30024906 PMC6053140

[mcn13034-bib-0010] Hofmeyr, G. J. , Lawrie, T. A. , Atallah, Á. N. , & Torloni, M. R. (2018). Calcium supplementation during pregnancy for preventing hypertensive disorders and related problems. Cochrane Database of Systematic Reviews, 10, CD001059 10.1002/14651858.CD001059.pub5 30277579 PMC6517256

[mcn13034-bib-0011] Ibitoye, M. , Choi, C. , Tai, H. , Lee, G. , & Sommer, M. (2017). Early menarche: A systematic review of its effect on sexual and reproductive health in low‐ and middle‐income countries. PLoS ONE, 12 10.1371/journal.pone.0178884 PMC5462398 28591132

[mcn13034-bib-0012] Inchley, J. , Currie, D. , Young, T. , Samdal, O. , Torsheim, T. , Augustson, L. , … Barnekow, V. (2016). Growing up unequal: Health Behaviour in School‐aged Children (HBSC) study: International report from the 2013/2014 survey. In *Health Policy for Children and Adolescents.* ISBN 987‐92‐890‐1423‐6

[mcn13034-bib-0013] Institute of Medicine . (2009). Weight Gain in Pregnancy: Reexamining the Guidelines. In *Human reproduction.* Retrieved from http://humrep.oxfordjournals.org/content/12/suppl_1/110.short

[mcn13034-bib-0014] Institute of Medicine , & Food and Nutrition Board . (2011). In Ross A. L. Y. C. A., Taylor C. L., & Del Valle H. B. (Eds.), DRI: Dietary reference intakes: Calcium and vitamin D. Washington (DC): National Academies Press http://www.nap.edu 21796828

[mcn13034-bib-0015] Kovacs, C. S. (2016). Maternal mineral and bone metabolism during pregnancy, lactation, and post‐weaning recovery. Physiological Reviews, 96, 449–547. 10.1152/physrev.00027.2015 26887676

[mcn13034-bib-0016] Lappe, J. M. , McMahon, D. J. , Laughlin, A. , Hanson, C. , Desmangles, J. C. , Begley, M. , & Schwartz, M. (2017). The effect of increasing dairy calcium intake of adolescent girls on changes in body fat and weight. The American Journal of Clinical Nutrition, 105(5), 1046–1053. 10.3945/ajcn.116.138941 28298396 PMC5402032

[mcn13034-bib-0017] Li, H. , Song, L. , Shen, L. , Liu, B. , Zheng, X. , Zhang, L. , … Xu, S. (2017). Age at menarche and prevalence of preterm birth: Results from the Healthy Baby Cohort study. Scientific Reports, 7 10.1038/s41598-017-12817-2 PMC5626706 28974739

[mcn13034-bib-0018] Maia, E. G. , da Silva, L. E. S. , Santos, M. A. S. , Barufaldi, L. A. , da Silva, S. U. , Claro, R. M. , … Claro, R. M. (2018). Padrões alimentares, características sociodemográficas e comportamentais entre adolescentes brasileiros. Revista Brasileira de Epidemiologia, 21(suppl 1), e180009 10.1590/1980-549720180009.supl.1 30517460

[mcn13034-bib-0019] Marangoni, F. , Pellegrino, L. , Verduci, E. , Ghiselli, A. , Bernabei, R. , Calvani, R. , … Poli, A. (2019). Cow's milk consumption and health: A health professional's guide. Journal of the American College of Nutrition, 38(3), 197–208. 10.1080/07315724.2018.1491016 30247998

[mcn13034-bib-0020] Monteiro, C. A. , Levy, R. B. , Claro, R. M. , de Castro, I. R. R. , & Cannon, G. (2010). A new classification of foods based on the extent and purpose of their processing. Cadernos de Saúde Pública, 26(11), 2039–2049. 10.1590/S0102-311X2010001100005 21180977

[mcn13034-bib-0021] Monzani, A. , Ricotti, R. , Caputo, M. , Solito, A. , Archero, F. , Bellone, S. , … Prodam, F. (2019). A systematic review of the association of skipping breakfast with weight and cardiometabolic risk factors in children and adolescents. What should we better investigate in the future? Nutrients, 11(2), 387 10.3390/nu11020387 PMC6412508 30781797

[mcn13034-bib-0022] Mousa, A. , Naqash, A. , Lim, S. , Mousa, A. , Naqash, A. , & Lim, S. (2019). Macronutrient and micronutrient intake during pregnancy: An overview of recent evidence. Nutrients, 11(2), 443 10.3390/nu11020443 PMC6413112 30791647

[mcn13034-bib-0023] O'Neil, C. E. , Byrd‐Bredbenner, C. , Hayes, D. , Jana, L. , Klinger, S. E. , & Stephenson‐Martin, S. (2014). The role of breakfast in health: Definition and criteria for a quality breakfast. Journal of the Academy of Nutrition and Dietetics, 114(12), S8–S26. 10.1016/J.JAND.2014.08.022 25458994

[mcn13034-bib-0024] Pinheiro, A. , Lacerda, E. , Benzecry, E. , Gomes, M. , & Costa, V. (2001). Tabela para avaliação de consumo em medidas caseiras (4th ed.). São Paulo: Atheneu.

[mcn13034-bib-0025] Pinho‐Pompeu, M. , Paulino, D. S. M. , Morais, S. S. , Crubelatti, M. Y. , Pinto Silva, J. L. E. , & Surita, F. G. (2019). How to classify BMI among pregnant adolescents? A prospective cohort. Public Health Nutrition, 22(2), 265–272. 10.1017/S1368980018002768 30378516 PMC10277188

[mcn13034-bib-0026] Pot, G. K. (2018). Sleep and dietary habits in the urban environment: The role of chrono‐nutrition. Proceedings of the Nutrition Society, 77(3), 189–198. 10.1017/S0029665117003974 29065932

[mcn13034-bib-0027] Rodrigues, P. R. M. , Luiz, R. R. , Monteiro, L. S. , Ferreira, M. G. , Gonçalves‐Silva, R. M. V. , & Pereira, R. A. (2017). Adolescents' unhealthy eating habits are associated with meal skipping. Nutrition, 42, 114–120.e1. 10.1016/J.NUT.2017.03.011 28596058

[mcn13034-bib-0028] Rohatgi, K. W. , Tinius, R. A. , Cade, W. T. , Steele, E. M. , Cahill, A. G. , & Parra, D. C. (2017). Relationships between consumption of ultra‐processed foods, gestational weight gain and neonatal outcomes in a sample of US pregnant women. PeerJ, 5, e4091 10.7717/peerj.4091 29230355 PMC5723430

[mcn13034-bib-0029] Sally, E. O. F. , Anjos, L. A. D. , Ramos, E. G. , Fonseca, V. M. , Silva, B. A. M. D. , & Wahrlich, V. (2018). Dietary intake of pregnant adolescents cared for in primary health care units of a Brazilian urban municipality. Nutrición Hospitalaria, 35(3), 596–605. 10.20960/nh.1412 29974768

[mcn13034-bib-0030] St‐Onge, M.‐P. , Ard, J. , Baskin, M. L. , Chiuve, S. E. , Johnson, H. M. , Kris‐Etherton, P. , & Varady, K. (2017). Meal timing and frequency: Implications for cardiovascular disease prevention: A scientific statement from the American Heart Association. Circulation, 135(9), e96–e121. 10.1161/CIR.0000000000000476 28137935 PMC8532518

[mcn13034-bib-0031] The Lancet . (2020). Preventing teenage pregnancies in Brazil. Lancet (London, England), 395(10223), 468 Elsevier. 10.1016/S0140-6736(20)30352-4 32061278

[mcn13034-bib-0032] United Nations Population Fund . (2013). UNFPA, motherhood in childhood: Facing the challenge of adolescent pregnancy. In *State of the World Population 2013.*

[mcn13034-bib-0033] Wallace, J. M. (2019). Competition for nutrients in pregnant adolescents: Consequences for maternal, conceptus and offspring endocrine systems. Journal of Endocrinology, 242, T1–T19. 10.1530/joe-18-0670 30615597

[mcn13034-bib-0034] Wise, N. J. (2015). Pregnant adolescents, beliefs about healthy eating, factors that influence food choices, and nutrition education preferences. Journal of Midwifery & Women's Health, 60(4), 410–418. 10.1111/jmwh.12275 26255801

